# Children’s dietary diversity and related factors in Rwanda and Burundi: A multilevel analysis using 2010 Demographic and Health Surveys

**DOI:** 10.1371/journal.pone.0223237

**Published:** 2019-10-09

**Authors:** Estefania Custodio, Zaida Herrador, Tharcisse Nkunzimana, Dorota Węziak-Białowolska, Ana Perez-Hoyos, Francois Kayitakire

**Affiliations:** 1 European Commission Joint Research Centre, Ispra, Italy; 2 Instituto de Salud Carlos III, Centro Nacional de Medicina Tropical, Madrid, Spain; 3 Sustainability and Health Initiative (SHINE), Department of Environmental Health, Harvard T. H. Chan School of Public Health, Boston, Massachusetts, United States of America; Bielefeld University, GERMANY

## Abstract

**Background:**

One of the reported causes of high malnutrition rates in Burundi and Rwanda is children's inadequate dietary habits. The diet of children may be affected by individual characteristics and by the characteristics of the households and the communities in which they live. We used the minimum dietary diversity of children (MDD-C) indicator as a proxy of diet quality aiming at: 1) assess how much of the observed variation in MDD-C was attributed to community clustering, and 2) to identify the MDD-C associated factors.

**Methods:**

Data was obtained from the 2010 Demographic and Health Surveys of Burundi and Rwanda, from which only children 6 to 23 months from rural areas were analysed. The MDD-C was calculated according to the 2007 WHO/UNICEF guidelines. We computed the intra-class coefficient to assess the percentage of variation attributed to the clustering effect of living in the same community. And then we applied two-level logit regressions to investigate the association between MDD-C and potential risk factors following the hierarchical survey structure of DHS.

**Results:**

The MDD-C was 23% in rural Rwanda and 16% in rural Burundi, and a 29% of its variation in Rwanda and 17% in Burundi was attributable to community clustering. Increasing age and living standards were associated with higher MDD-C in both countries, and only in Burundi also increasing level of education of the mother's partner. In Rwanda alone, the increasing ages of the head of the household and of the mother at first birth were also positively associated with it. Despite the identification of an important proportion of the MDD-C variation due to clustering, we couldn't identify any community variable significantly associated with it.

**Conclusions:**

We recommend further research using hierarchical models, and to integrate dietary diversity in holistic interventions which take into account both the household's and the community's characteristics the children live in.

## Background

Adequate nutrition during infancy and early childhood is fundamental to the development of each child’s full human potential [1]. Poor infant feeding practices, coupled with high rates of infectious diseases, are the principal proximate causes of malnutrition during the first two years of life [2]. In turn, this period of life is a “critical window” for the promotion of optimal growth, health and behavioural development [1]. The UNICEF causal analysis framework identifies inappropriate dietary intakes and diseases as the *immediate causes* of children's malnutrition and mortality. These would consecutively be determined by the so-called *underlying causes*, grouped in caring practices, food security, and health services. These, in turn, would be grounded on the *basic or structural causes* like poverty, education, and lack of basic community resources, among others [3].

According to the Burundi´s and Rwanda´s Demographic and Health surveys (2010), around 58% and 44% of the country’s children were stunted, respectively [4,5]. In Rwanda, there have been important improvements in nutritional status of children under five years: the percentage of stunted children fell from 51% in 2005 to 44% in 2010 and 38% in 2014 [6]. On the other hand, Burundi has the highest rate of stunting in East Africa, reaching in 2010 the highest percentage of stunted children in the last two decades (58%)[7], which declined to 56% in 2016 [8]. In both countries, the underlying and basic causes of malnutrition are poor socio-economic and education status of the household with very low purchasing power, agricultural market dysfunctions, poor infrastructures, decline of the per capita food production and other institutional and organizational failures [4,5,9].

However, the direct causes related to dietary inadequacy are poorly understood in these populations. One of the proxies for dietary adequacy in children is the diet diversity (DD), referring to the consumption of foods from the major nutritionally important types of food, while providing some balance between plant foods and animal-source foods. More diverse diets according to this definition have shown to be associated with an improved diet in terms of micronutrients, and to improved nutritional status among children 6–23 months old [10,11]. In fact, according to the World Health Organization (WHO) guidelines, the DD of children 6–23 months of age, is one of the core indicators for adequate nutrition during infancy and early childhood [1].

The dietary intakes of children may be affected by their age, gender and health status due to physiological conditions or cultural patterns associated with these individual characteristics. The household food access, the intra-household distribution of foods and the care giver's behaviours based on culture, perceptions and societal conventions, among others may impact the children's diet at the household level[10]. Finally, community characteristics like the food availability and the stability of the food supply [12], determined by biophysical characteristics of the environment and by the resource infrastructures, may also play a role on the foods consumed by young children.

In this paper, we aimed firstly to assess how much of the variation in the DD of the 6–23 months old children in rural Burundi and rural Rwanda was attributed to individual or household characteristics of the children, and how much to community characteristics. Secondly, we aimed to identify which of those characteristics were significantly associated with the minimum dietary diversity (MDD) of those children using a multilevel strategy.

The importance of this study relies on being the first to assess and compare children's dietary diversity in Rwanda and Burundi. Also, it is also among the firsts to apply a multilevel modelling to the analysis of children's dietary diversity including biophysical variables derived from environmental datasets, thus applying a multidisciplinary approach to the analysis.

## Methods

### Study area and population

The republics of Rwanda and Burundi are landlocked countries situated in central Africa. Both are considered to be among the smallest and most densely populated countries in Africa (12.3 million people and 499 persons per km^2^ and 11.2 million people and 435 persons per km^2^, for Rwanda and Burundi respectively, in 2018)[13]. The proportion of women to men is 48% to 52% in both countries and the percentage of children below 14 years is 44% in Burundi and 39% in Rwanda[14] [15]. The two countries have a tropical climate, with moderate temperatures due to the high altitude which averages 1700 m above the sea level, and with two rainy seasons (September to December, and March to May) [16].

In Rwanda, agriculture is the main economic activity with around 72% of the working population employed in agriculture. Farmers practice mixed farming that combines rain fed root and tuber crops, cereals, dry beans, plantain, banana and traditional livestock-rearing with some vegetable production [17]. About 71% for all crops produced are consumed and only 23% are sold on the market [5] [18].

Agriculture is the backbone of Burundi’s economy, with 90% of the population depending on it for their livelihood [19]. The main staple crops grown are banana, cassava, sweet potato and beans. Common cash crops include coffee, cotton, sugar and tea. Most households’ farms produce little for the market, and yields are often not enough to meet their own needs. The long period of conflict had an adverse impact on the agriculture sector, including the livestock sector [20].

### Data collection and management

Nutritional status and socioeconomic information was obtained from the Demographic and Health Surveys (DHS), funded by the US Agency for International Development (USAID). DHS are cross-sectional household surveys that provide a nationally representative sample with a wide variety of information regarding household socioeconomic status, health access and behaviour, and nutrition in most developing countries in the world. DHS are based on a stratified two-stage sampling strategy. In the first stage, primary sampling units or clusters which correspond to villages or communities are selected from a frame list with probability proportional to a size measure. In the second stage, around 20–30 households are randomly selected and interviewed [21]. These data sets are in the public domain and are available from the DHS program web-site (https://www.dhsprogram.com/). In most countries, between 3,000 and 10,000 children below the age of 60 months are assessed for their growth status using anthropometric measurements, but not all surveys collect the dietary questionnaire for children 6–23 months old [22].

For this study, the sample was limited to households with children aged 6–23 months old with no missing observations on DD. We analysed only rural data as the resulting urban samples (Rwanda n = 161 and Burundi n = 222) had few observations per community rendering robustness of results of two-level modelling questionable. There were a total of 2014 children 6 to 23 months in rural Burundi and rural Rwanda (N = 961 and N = 1053 respectively) which after restriction of valid data entries resulted in N = 2,006 (Rwanda n = 1049 and Burundi n = 957). Sampling weights were employed to get more precise estimates of parameters. And in order to correct standard errors of estimates resulting from complex sample design, we accommodated clustering (primary sampling units) and stratification in the analysis.

DHS collect data via face-to-face interview which are conducted by a same-sex interviewer. They utilize standard core questionnaires household, women's and oftentimes men's, to ensure comparability across countries and over time. The household questionnaire lists all members in the household and collects information of the dwelling itself, such as the source of water, type of sanitation facilities, ownership of various consumer goods, etc. The Woman's questionnaire contains information on several topics concerning background characteristics, health and reproductive behaviour as well as information on children's delivery, postnatal care and feeding and caring practices, among others. The children's dietary data is collected by using adapted food groups check lists based on food consumed on the previous 24 hours. Detailed information on DHS questionnaires can be found at https://dhsprogram.com/What-We-Do/Survey-Types/DHS%20Questionnaires.cfm#CP_JUMP_16179

Fieldwork in Rwanda was conducted from September 26, 2010 to March 10, 2011, and in Burundi from August 29, 2010 to January 30, 2011 in Burundi. These time periods correspond to part of the short dry and the short rainy seasons in both countries[23] [24].

### Variables

#### Outcome variables

The dietary diversity indicator used in the analysis was created using data from the 24 hour recall of the food groups (FG) available in the DHS surveys [21,22].

As recommended for the calculation of the dietary diversity score (DDS-7) for children 6–23 months of age, the food groups collected by the DHS questionnaire, were rearranged in 7 food groups: 1-Grains, roots and tubers, 2-Legumes and nuts 3-Dairy products (milk, yogurt, cheese), 4-Flesh foods (meat, fish, poultry and liver/organ meats), 5-Eggs, 6-Vitamin-A rich fruits and vegetables and 7-other fruits and vegetables. We constructed the dichotomous minimum dietary diversity for children (MDD-C) variable by computing 1 if the child had a DDS-7 equal to or higher than 4, and a 0 if his/her DDS-7 was below 4.

#### Covariates

**Fig 1:**
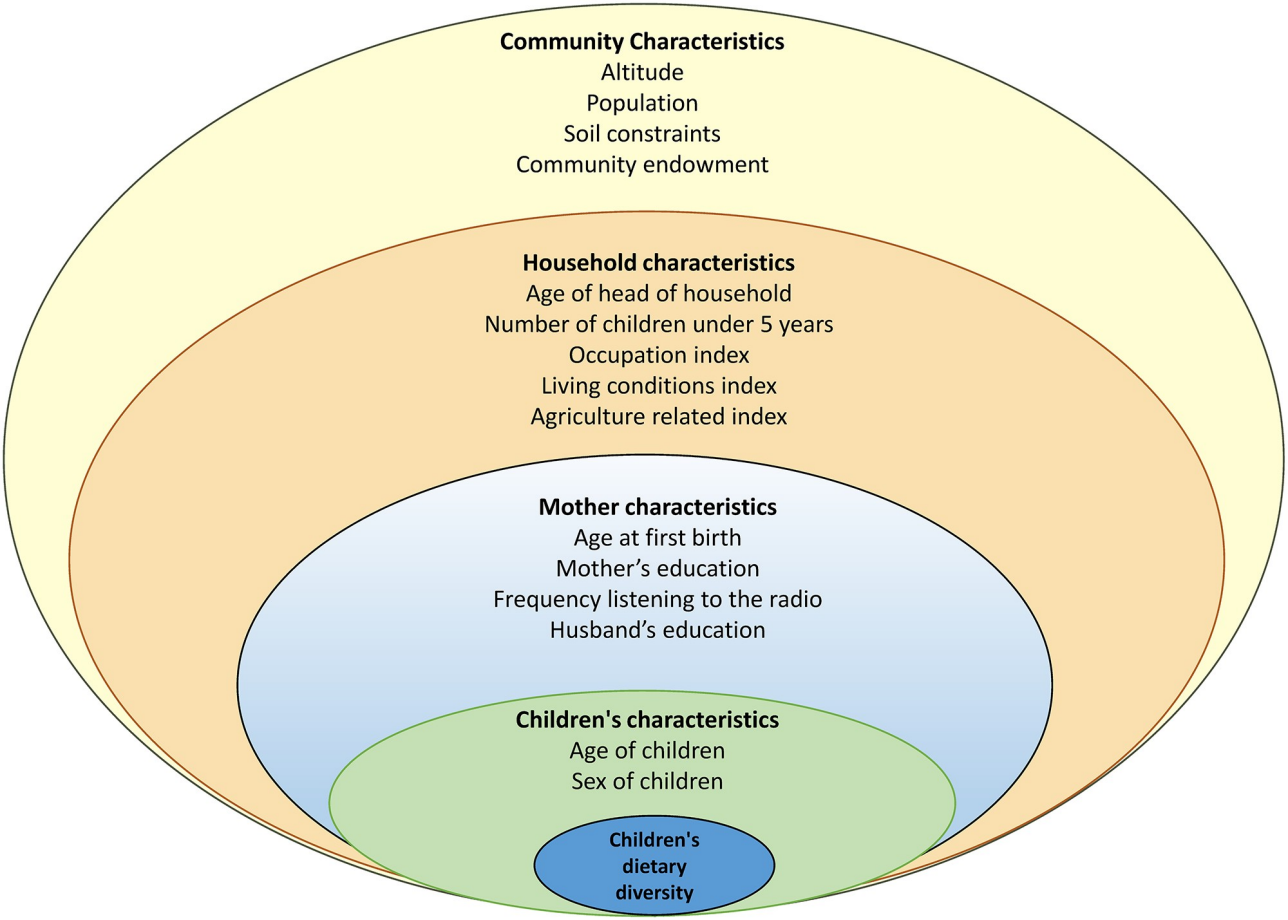
Conceptual framework, Rwanda and Burundi DHS, 2010.

We built our conceptual framework ("Fig 1") based on a literature review of the variables which have already shown a relevant role in children's diet and/or nutritional status. These variables encompass factors at child, maternal, household and community level which can impact children's dietary intake as described in the Introduction.

Moreover, the conceptual framework reflects the hierarchical nature of data as due to the stratified nature of data in the DHS, children are nested into mothers, mothers are nested into households and households into communities ("Fig 1").

The socioeconomic and health information at individual, household and community level was obtained from the DHS, while the biophysical characteristics of the community (altitude, population and soil constraints) were obtained from existing environmental datasets.

#### Composite indicators

We constructed four composite indicators for the socioeconomic characteristics at household level and one at the community level.

The composite indicators for socioeconomic characteristics at household level were calculated through categorical principal component analysis (CATPCA). This procedure simultaneously quantifies categorical variables while reducing the dimensionality of the data. Standard principal components analysis assumes linear relationships between numeric variables. On the other hand, the optimal-scaling approach allows variables to be scaled at different levels. Categorical variables are optimally quantified in the specified dimensionality. As a result, nonlinear relationships between variables can be modelled [25].

Based on the results of CATPCA (analysis of eigenvalues and patterns of loadings) four composite indicators were calculated: the educational index to assess the educational level of the mother and partner, the occupation index to assess labour market related situation of the household, the living conditions index and the agricultural index. The latter two assess the household wealth situation separating agricultural related proxies and non-agricultural related. See description of the variables used for the construction of each index in S1 Table. All indices were oriented the higher, the better.

To assess the access to services in communities, the community endowment index was calculated. Three indicators (% of households having improved water in a community (I1); % of households having electricity in a community (I2); and % of households having land-line phone in a community (I3)–calculated for each community from the DHS) were used. They were aggregated using a generalised mean of power 0.5. The choice of generalised mean of power 0.5 (instead of, for example, simple arithmetic average) was motivated by our belief that a community to score well should ensure good access to all services. It implies that an improvement in one variable cannot fully compensate for equal deterioration in another variable. This aggregation method ensures that the compensation of low results in one dimension with high results in others is only partial [26]. Higher values of the index indicated better access.

Values of all five indices were categorized into one of three categories: low, medium, high according to tertiles.

#### Biophysical indicators (remote sensing)

The potential landscape drivers derived from environmental datasets, which can influence health and nutrition outcomes were identified based on literature [27]. These variables were matched with the DHS datasets through the GPS coordinates provided in the survey for the centre of the populated area surveyed (cluster centroid). GPS data is subject to a standardized geographical displacement procedure that ensures anonymity of respondents, meaning that GPS points are randomly displaced up to 5 km and up to 2 km in urban areas. A further 1% of the rural sample points are offset up to 10 km [28]. To deal with spatial inconsistences between the GPS and the biophysical variables, a buffer of 10-km radius around the centroid was created and the value of each cluster was computed as the average of the value within the radius [29].

Population density was derived from the UN-Adjusted Gridded Population of the World (GPW) v4 for year 2010. Available at http://ghdx.healthdata.org/record/gridded-population-world-version-4-un-adjusted-population-count-2000-2005-2010-2015-2020

The mean altitude of each cluster was retrieved from the Global Multi-Resolution Terrain Elevation Data 2010 (GMTED2010) at 7.5 arc-second spatial resolution (approximately 225m/grid), developed by the U.S. Geological Survey (USGS), the Earth Resources Observation System Data Center and the National Geospatial Intelligence Agency (NGA) [30].

We used the global land area with soil constraints developed by the Food Insecurity, Poverty and Environment Global GIS Database to count for the effect of soil properties on agricultural production. The dataset combines soil depth and quality, and other soil characteristics to establish the severity of the constraints combining data from 2000 to 2007 [31].

The Normalized Difference Vegetation Index (NDVI) data derived from NASA’s Moderate Resolution Spectroradiometer at 250 m spatial resolution[32]. NDVI quantifies the concentrations of green leaf vegetation; in this study, the long-term average (2001–2011) from June to August is computed to characterize vegetation status during the dry season.

#### Ethical considerations

Procedures and questionnaires for standard DHS surveys have been reviewed and approved by ICF Institutional Review Board, ensuring that the survey complies with the U.S. Department of Health and Human Services regulations for the protection of human subjects (45 CFR 46).

Before each interview an informed consent statement is read to the respondent, who may accept or decline to participate.

### Data analysis

We used descriptive and analytic statistical methods to present the findings of this study. Frequencies and cross tabulation were used to summarize descriptive data statistics in tables and figures.

We computed the intra-class correlation coefficient (ICC) in order to identify how much variation in the dietary diversity was attributed to (1) individual/household characteristics of children and their mothers and (2) to community characteristics.

We applied two-level logit regressions to simultaneously investigate the association between potential risk factors and the MDD-C in all eligible children under the hierarchical survey structure of DHS. We aimed to generate robust standard errors around estimates of association on two levels of factors: individual or household level and community level factors. We first estimated an ‘‘empty” model (model 0), which only includes a random intercept and allowed us to detect the existence of a possible contextual dimension for this phenomenon [33]. Thereafter, we included the individual, mother and household characteristics in the model (model 1) to investigate the extent to which MDD was explained by these characteristics. To identify the variables to be introduced in the models we first carried out bivariable regressions with the outcome of interest. The variables that were significant at the 0.1 level were then introduced in the multivariable. For final models we kept all the variables that were significant at the p<0.05 in the any of the multivariable models. Household level was not assessed separately because of seldom households with more than 1 child aged 6–23 months. Finally we added the community variables (model 2) to investigate whether the diet diversity was conditioned by specific community characteristics.

The adjusted odds ratios (aOR) with the 95% confidence intervals (95% CI) were computed to assess the associated factors with MDD-C. To compare the individual level and community level effects on DD the median odds ratio (MOR) were calculated. Log likelihood ratio tests were used to compare null models with single-level and multi-level models by using the *melogit* STATA function. Statistical analyses were performed using STATA software, version 15.0.

## Results

The study included a total of 957 and 1049 children aged 6–23 months from rural Burundi and rural Rwanda, respectively. The MDD-C was 23% in Rwanda compared to 16% in Burundi (p<0.001). The mean age of the respondent women at first birth was significantly lower in Burundi than in Rwanda (p<0.001). Also the education level of the women and their partners was significantly lower in Burundi as compared to Rwanda (p<0.001). Severe soil constraints were observed and associated to Burundian households more frequently to the Rwandan households (87.5% vs. 48.5%, respectively) by the spatial analysis. Other differences between the two countries are summarized in Table 1.

**Table 1:** Comparison of Burundi's and Rwanda's sample populations. DHS 2010.

COVARIATES		BURUNDI (N = 957)		RWANDA (N = 1049)		
Variables	Categories	n	%	n	%	p*
**Minimum dietary diversity for children**	**Yes**	151	15.8	241	23.0	**<0.0001**
**Sex of the child**	**Female**	486	51.0	525	50.0	**0.71**
**Age of the child**	6–11 months	316	33.0	353	33.7	**0.838**
	12–17 months	324	33.9	342	32.6	
	18–23 months	317	33.1	354	33.8	
**Age of mother at 1st birth**	**12–18 years**	241	25.2	165	15.7	**<0.0001**
	19–24 years	629	65.7	702	66.9	
	25–37 years	87	9.1	182	17.4	
**Mother's education**	**No education**	538	56.2	189	18.1	**<0.0001**
	primary	380	39.7	794	15.7	
	secondary and higher	39	4.1	66	6.3	
**Mother's partner's education**	**No education**	386	40.3	188	17.9	**<0.0001**
	Primary	487	50.9	688	56.6	
	Secondary or higher	84	8.8	173	16.5	
**Mother's partner's occupation**	**Agriculture related**	685	73.3	738	70.4	**0.151**
**Age of head of the household**	**Below 31**	427	44.6	429	40.9	**0.269**
	Between 30 and 40	271	28.3	315	30.0	
	between 40 and 50	169	17.7	186	17.7	
	More than 50	90	9.4	119	11.3	
**Frequency of listening to radio in the household**	**less than once a week**	437	45.7	403	38.5	**0.001**
	at least once a week	519	54.3	644	61.5	
**Number of children under 5 in the household**	**One**	245	25.7	390	37.3	**<0.0001**
	Two	542	56.8	531	50.8	
	More than 2	167	17.5	124	11.9	
***Altitud (mean)***		*1551 mts*		*1745 mts*		
**Soil constrains**	**No soil constraints**	0	0	83	7.9	**<0.0001**
	Partial soil constraints	119	12.4	455	43.4	
	Frequent soil constraints	317	33.1	283	27.0	
	Very frequent soil constraints	521	54.4	228	21.7	

* Chi^2^ test applied to the comparison of Burundi and Rwanda's distribution of selected variables.

**Fig 2:**
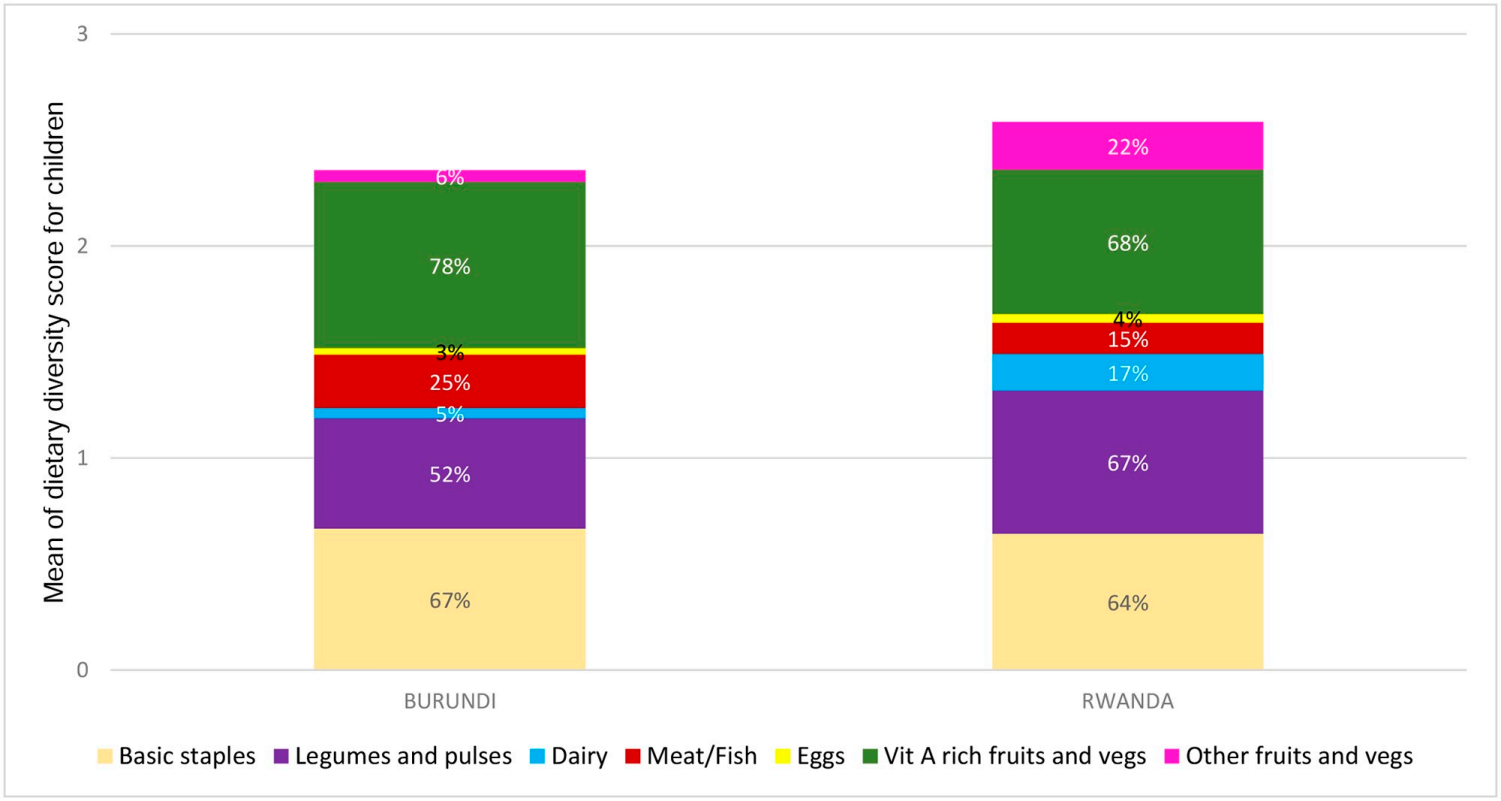
Mean dietary diversity and food groups’ consumption in children aged 6–23 months in Burundi and Rwanda, DHS 2010.

The mean of the dietary diversity score for children was 2.4 in Burundi and 2.6 in Rwanda. Flesh foods and vitamin A rich fruits and vegetables were consumed in higher proportion by children in Burundi while other fruits, dairy and legumes and pulses were more frequently consumed by Rwandan children. Eggs consumption was below 5% in both countries, while the most consumed group in both countries was vitamin A rich fruits and vegetables, followed by basic staples and legumes and pulses. Flesh food consumption was higher in Burundi (25%) than in Rwanda (15%), whereas dairy consumption was considerably higher in Rwanda (17%) as compared to 5% in Burundi ("Fig 2").

**Fig 3:**
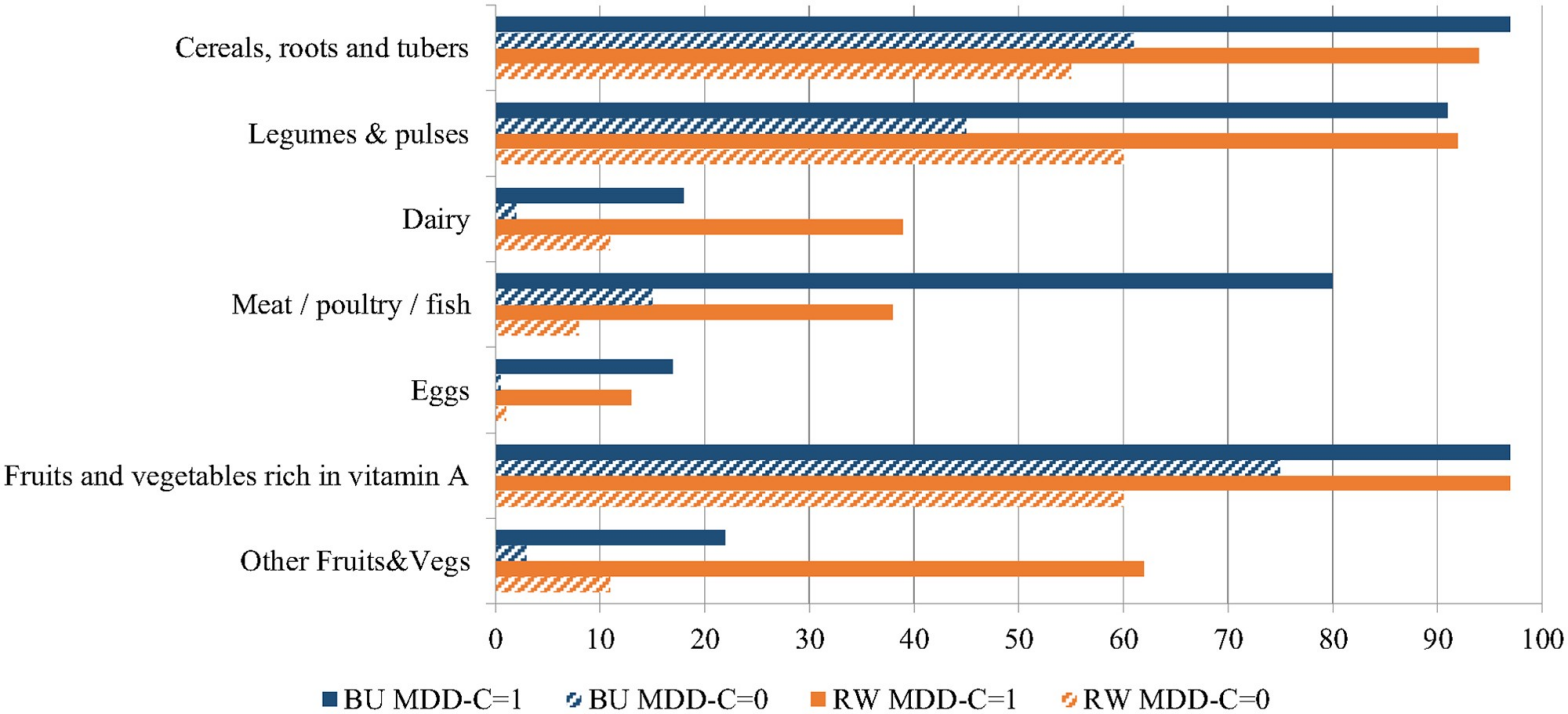
Percentage of children aged 6–23 months who consume each food group by MDD in Burundi and Rwanda, DHS 2010.

In Fig 3 we can observe the differences in food groups’ consumption by MDD-C status. Among children who met the MDD-C criterion, consuming at least four different food groups, the most common combination of FG was basic staples, legumes and pulses, and fruits and vegetables rich in vitamin A in both countries plus an alternative FG (flesh foods more frequently in Burundi while other fruits and vegetables in Rwanda). In Burundi there was a considerable difference between the children reaching the MDD-C and those children not reaching it in the consumption of flesh foods (80% vs. 18%, respectively). The difference between these two groups was not so marked in Rwanda, where those foods also showed a lower frequency of consumption. In Burundi, the difference by MDD-C status was also specially marked for other foods from animal sources like dairy and eggs, while in Rwanda there were also relevant differences in the consumption of groups of vegetable origin like legumes and pulses, cereals and roots or other fruits and vegetables. The consumption of eggs was below 20% in all the groups, between 12 and 18% among children consuming four or more food groups and almost insignificant among children not reaching the MDD-C ("Fig 3").

As indicated in Table 2, from the total variation in MDD-C across communities, 29.2% (95% CI: 16.5%–46.2%) in Burundi and 17.1% (95% CI: 9.7%–28.3%) in Rwanda was attributable to clustering (to the fact that children and their families living in the same community are likely to be more alike in terms of DD than children/families from different communities), which suggests the need for multilevel mixed effects regression analysis rather than using the traditional (one level) regression analysis.

**Table 2:** Model estimates for factors associated with MDD-C in Burundi and Rwanda DHS2010.

**BURUNDI**	**Model 0**	**Model 1**	**Model 2**
Log likelihood (LL)	-452.66678	-424.2404	-420.56354
Error variance at community level	1.36	1.61	1.46
Intra-class correlation coefficient (ICC)	29.2%	32.8%	30.7%
Median odds ratio (MOR)	3.0	3.3	3.2
**RWANDA**	**Model 0**	**Model 1**	**Model 2**
Log likelihood (LL)	-576.89601	-536.30295	-533.33558
Error variance at community level	0.68	0.60	0.56
Intra-class correlation coefficient (ICC)	17.1%	15.4%	14.5%
Median odds ratio (MOR)	2.2	2.1	2.0

Also, the explanatory power of our models improved when children/household variables as well as community variables were introduced, as shown by increasing LL between M0, M1 and M2, for both Burundi and Rwanda.

Adjusted odds ratios (95% CI) for rural Burundi and rural Rwanda are shown in Tables 3 and 4 respectively. Among the individual and household level factors, the odds of meeting the MDD-C criterion was significantly higher among older children, by more than two folds for children older than one year old as compared to infants. Also in both countries, children living in households with higher living standards index were more likely to meet the MDD-C criterion. In Burundi, the higher level of education of the mother's husband/partner's was also associated with higher MDD. On the other hand, in Rwanda, higher ages of the head of the household and of the mother at the first birth were positively associated with meeting MDD-C. The mothers’ educational level and the educational index were positively associated with MDD-C in both countries, but only in the bivariable model (S2 Table), and lost significance when the living standards index was introduced.

**Table 3:** Factors associated with MDD-C in rural Burundi DHS2010. Results from the two-level logit regression model.

		M0 (empty model)	M1 (individual)	M2 (ind + community)
Variables	Categories	AOR (95% CI)	AOR (95% CI)	AOR (95% CI)
**Sex of the child**	**Male**	**Reference**	**Reference**	**Reference**
	Female		1.4 (0.9–2.2)	1.4 (0.9–2.2)
**Age of the child**	**6–11 months**	**Reference**	**Reference**	**Reference**
	12–17 months		2.5 (1.4–4.5)	2.5 (1.4–4.5)
	18–23 months		1.9(0.9–2.2)	1.9(0.9–2.2)
**Age of mother at 1st birth**	**12–18 years**	**Reference**	**Reference**	**Reference**
	19–24 years		0.9(0.5–1.5)	0.9(0.5–1.6)
	25–37 years		0.9(0.4–2.2)	1.0(0.4–2.3)
**Mother's partner's education**	**No education**	**Reference**	**Reference**	**Reference**
	Primary		2.2(1.3–3.7)	2.2(1.3–3.8)
	Secondary or higher		3.7(1.6–8.3)	3.8(1.5–8.9)
**Age of head of the household**	**Below 31 years**	**Reference**	**Reference**	**Reference**
	Between 30 and 40 years		1.5(0.9–2.7)	1.5(0.8–2.6)
	between 40 and 50 years		1.3 (06–2.5)	1.3 (0.6–2.4)
	More than 50 years		1.3(0.5–3.2)	1.4(0.6–3.3)
**Number of children under 5 in the houeshold**	**One**	**Reference**	**Reference**	**Reference**
	Two		1.1(0.6–2.0)	1.1(0.6–2.0)
	More than 2		1.0(0.5–2.2)	1.0(0.5–2.3)
Living conditions index*	**Low**	**Reference**	**Reference**	**Reference**
	Middle		2.4(1.2–4.6)	2.3(1.2–4.3)
	High		3.1(1.6–6.0)	3.8(1.6–5.8)
Community Endowment index*	**Low**	**Reference**	**Reference**	**Reference**
	Medium			0.9(0.5–1.7)
	High			0.8(0.4–1.5)
**Soil Constraints score**§	**Partial constraints**	**Reference**	**Reference**	**Reference**
	Frequent Severe constraints			1.6 (0.6–4.7)
	Very frequent severe constraints			1.1(0.4–2.7)
***Altitud (continuous)***				*1*.*0(1*.*0–1*.*0)*
***Population (continuous)***				*1*.*0(1*.*0–1*.*0)*

* Indices created by principal component analysis (See Methods section)

^§^Burundi did not have any communities identified as with no soil constraints, therefore the reference becomes the partial constraints category.

**Table 4:** Factors associated with MDDw in rural Rwanda DHS2010. Individual and household variables.

COVARIATES				
		M0 (empty model)	M1 (individual)	M2 (ind + community)
Variables	Categories	AOR (95% CI)	AOR (95% CI)	AOR (95% CI)
**Sex of the child**	**Male**	**Reference**	**Reference**	**Reference**
	Female		1.2 (0.9–1.6)	1.2(0.9–1.6)
**Age of the child**	**6–11 months**	**Reference**	**Reference**	**Reference**
	12–17 months		2.0(1.3–3.1)	2.0(1.3–3.1)
	18–23 months		2.1(1.4–3.3)	2.1(1.4–3.3)
**Age of mother at 1st birth**	**12–18 years**	**Reference**	**Reference**	**Reference**
	19–24 years		2.0(1.2–3.4)	1.9(1.2–3.2)
	25–37 years		2.4(1.2–4.5)	2.3(1.2–4.3)
**Mother's partner's education**	**No education**	**Reference**	**Reference**	**Reference**
	Primary		0.9(0.6–1.6)	0.9(0.6–1.6)
	Secondary or higher		1.6(0.9–2.8)	1.6(0.9–2.8)
**Age of head of the household**	**Below 31 years**	**Reference**	**Reference**	**Reference**
	Between 30 and 40 years		2.0(1.3–3.0)	2.0(1.3–3.0)
	Between 40 and 50 years		1.8(1.1–3.2)	1.8(1.0–3.1)
	More than 50 years		1.6(0.9–2.8)	1.6(0.9–2.8)
**Number of children under 5 in the houeshold**	**One**	**Reference**	**Reference**	**Reference**
	Two		0.7(0.5–0.9)	0.7(0.5–0.9)
	More than 2		0.7(0.4–1.2)	0.6(0.4–1.1)
Living conditions index*	**Low**	**Reference**	**Reference**	**Reference**
	Medium		1.1(0.7–1.7)	1.1(0.7–1.7)
	High		2.3(1.5–3.5)	2.3(1.5–3.5)
Community Endowment index*	**low**	**Reference**	**Reference**	**Reference**
	Medium			1.0(0.7–1.6)
	High			1.3(0.8–1.9)
**Soil Constraints score**	**No constraints**	**Reference**	**Reference**	**Reference**
	Partial constraints			1.3(0.6–2.6)
	Frequent severe constraints			1.0(0.5–2.0)
	Very frequent severe constraints			0.8(0.4–1.9)
***Altitud (continuous)***				*1*.*0 (1*.*0–1*.*0)*
***Population (continuous)***				*1*.*0(0*.*7–1*.*6)*

* Indices created by principal component analysis (See Methods section)

The agricultural wealth index was only significant in the bivariable model for Rwanda (S2 Table). None of the community level variables were significantly associated with MDD-C in neither country (S3 Table). Moreover, community covariates didn´t influence the associations previously described.

## Discussion

The first 1000 days (from conception to a child’s second birthday) is a critical period for human health and development, of which the benefit could last throughout life. According to the 2010 DHS, the percentage of infants < 6 months old exclusively breastfed in Rwanda and Burundi was 83.8 and 69.3% respectively, and 93% in the two countries for continued breastfeeding at 1 year of age. These data represent a significant progress compared to previous years. Moreover, these figures are higher than those seen in neighbouring countries [34].

For the complementary feeding period, our results show that MDD-C was met by 23% and 16% of children aged 6–23 months in rural Rwanda and rural Burundi, respectively. Regarding the figures from other Subsaharan countries, in the same year the percentage of children with optimum MDD-C in Ethiopia (4%), and Zimbabwe (14%) were lower than Burundi while Malawi (26%) and Tanzania (23%) were higher than Rwanda [34]. However, for both countries the latest figures show slight improvement in recent years, with rural Rwanda having increased the MDD-C to 27%% in 2014 and Burundi to 17% in 2016 [6,8]. Moreover, is important to note that in these two countries the MDD-C reported for urban settings is much higher as compared to rural, reaching 47% in Rwanda and 35% in Burundi in year 2010 [23] [24]. This positive difference among the urban populations has already been described in other African settings and has been associated with differences in household wealth and parental education, as well as unequal access to health care[35].

The food groups consumed were broadly similar in both countries, mainly cereals and roots, legumes and pulses, and fruits and vegetables rich in vitamin A. However, unlike findings in other settings, the basic staples food group was not the most frequently consumed (with a frequency ranging 60–70%), but rather the Vit A rich fruits and vegetables with a frequency of 78% in Burundi and 68% in Rwanda.

The main difference between the two countries was that flesh foods consumption was more frequent in Burundi while in Rwanda other fruits and vegetables were more common in the children's diets. Only 15% of Rwandan children had consumed flesh foods (meat/poultry or fish) the day before the survey, which is a low figure compared to small scale surveys in other countries like Madagascar with 55% of flesh food consumption [36], or the field surveys conducted by the German development agency (GIZ) which reported flesh consumption among children 6–23 months of age above 20% in the study settings of Zambia (21%), Malawi (25%), Benin (31%), Burkina Faso (48%), Togo (63%) and Mali (92%) among others [37]. In Rwanda, food supply of animal products has been reported to be very limited, mainly due to agro-ecological constraints [38]. On its progress towards the Comprehensive Africa Agriculture Development Programme implementation, the Government of Rwanda has recognized the central role of the animal production, targeting this key problem through the progressive modernizing of traditional livestock and the expansion of land area that is reserved for pasture [39]. Initiatives like the Rwanda Livestock Master Plan have recently accounted for improvement overtime [38]. In Burundi, around 70% of the households own some type of small livestock, whereas fewer households own cows [40]. The consumption of dairies reported was much lower in Burundi than in Rwanda, probably related to the implementation of specific programmes, namely the “Girinka” (One Cow Per Poor Family) programme, initiated since 2006 and aiming at increasing children's milk consumption in Rwanda [41].

Eggs consumption was particularly low in both countries. Intake of eggs has been reported to be very low in similar contexts [37] despite the fact that many African households raise poultry. In most African countries, the poultry is raised mainly for meat. The local chicken breeds have very low egg productivity. Moreover, animals are considered as assets, thus sold instead of being consumed [42]. Behavioural and cultural differences may also account for the low consumption [43]. In order to successfully address the low consumption of dairy, flesh foods and eggs, further investigation on the barriers and drivers of food from animal source consumption in both countries is needed.

Increasing child’s age was significantly associated with MDD-C in both countries. Previous studies have found that younger children were significantly associated with inadequate DD; and it has been related to the delay in the initiation of complementary feeding in the form of solid, semi-solid or soft foods [44–47]The living conditions index, which encompasses access to services, housing conditions and possessions of durables like radio, TV and refrigerator, was also positively associated to meet the MDD-C in both countries. It may be a proxy for wealth reflecting the improved access to foods purchased outside the household. Households assets have been related to MDD-C in previous studies [36,48]. Moreover, members of households with higher level of living standards index have probably better access to nutritional and health information. The index for agricultural related wealth was significant only in Rwanda in the bivariable analysis and lost significance in the multivariable model when the living conditions index was introduced, suggesting that living conditions overall had a greater impact than the ownership of agricultural land or livestock on the children's dietary diversity. However, studies conducted in Ethiopia, Malawi and Zambia have shown that agricultural related activities like crop and/or production diversification have been positively associated with child dietary diversity [49–51]Mother’s age at first birth and the age of the head of the household were positively associated with MDD-C in rural Rwanda. Increasing age of mothers at first birth may be reflecting the risk for school dropping and inappropriate children's feeding practices for women who become mothers at a very early age [52]. In Rwanda, older head of households may reflect better status, higher media exposure and better service utilization.

In our study, the mothers´ educational level was significantly associated to the MDD-C in the bivariable models of both countries, but lost significance when the living standards index was introduced in the multivariable model. It is well-established that mothers´ education has a positive effect on child nutrition in developing countries. More educated women shown better skills to access modern health services and for understanding health messages [53] and has been related to higher MDD before [36,54]. In our study samples, probably the mothers´ education is a protective factor in the poorest households, while loses relevance when the household incomes are high enough to meet the children daily requirements. However, in rural Burundi, the mother's partner's education was significantly associated with reaching MDD-C even after adjusting for the household living standards, suggesting that the level of education of the husband had an effect on the child's dietary diversity independently of the household wealth. There is ample evidence in the literature showing that children whose parents have a lower level of education are at higher risk of not meeting the requirement for MDD, which is consistent with this finding [44,48,55].

Around 17% and 28% of the variation in MDD-C in Rwanda and in Burundi, respectively, was determined by the fact that children living in the same communities are more alike in terms of dietary diversity than children living in different communities, suggesting that in order to improve dietary diversity of children, interventions should aim at targeting community potential constraints, and go beyond the individual and/or household targeting.

However, we failed to identify the community variables that may be responsible for the explanation of these percentages of variation in MDD-C. None of the community level variables we introduced in the model were significant. We recommend to perform multivariable analysis and explore other community variables which may impact children's dietary diversity, like the access to health and nutrition services[56] [57]or proximity and access to markets [51,58] which has proven to be positively associated with dietary diversity in other settings.

### Strengths and limitations

Our study had a number of strengths. Firstly the use of the Demographic Health Surveys which are nationally representative surveys using standardized methods, with data collected in the same year for both countries, which facilitates the interpretation of comparison. Secondly, the elaboration of new variables applying the principal component analysis and the calculation of biophysical and landscape-related variables linked to the DHS community by geospatial positioning, and thirdly the use of a multilevel analysis to account for contributors to the children's dietary diversity at the individual, household and community levels. Moreover, our results justified the use of multilevel analysis to differentiate the effects of variables at individual/household and community levels.

However, the cross-sectional nature of the surveys does not allow for casual interpretation of the determinants. Also, although variables at individual and household level were comprehensively collected in the DHS survey, sampling size did not allow for carrying out the multilevel analysis on urban populations and thus only rural ones were included. The sample size also limited the possibility to stratify the analysis by breastfeeding status, as 93% of children in each country were breastfeeding at the time of the survey. However, the similarity between countries on current breastfeeding and continued breastfeeding at 1 year prevalences allowed for the comparison of the overall MDD-C outcome (not stratified by breastfeeding status) between countries.

Finally, the variables obtained from remote-sensing were few, and did not capture the cluster variability observed on the outcome of interest, the minimum dietary diversity for children.

## Conclusions

The minimum dietary diversity for children (MDD-C) was low in both countries, particularly in Burundi. The household living standards were consistently related to improved children's dietary diversity, but other sociodemographic variables impacted MDD-C differently in each country.

In rural Rwanda and rural Burundi, part of the variability of the children's DD was explained by common characteristics of the communities the children lived in, which should be further explored to better understand the community effect.

We encourage research on diet diversity using multilevel analysis to identify potential drivers at community level.

Dietary diversification accounts for only one of the diet adequacy's dimensions. We recommend its inclusion in the strategies aiming to improve children's diet, by using an integrative approach which takes into account both the household's and the community's characteristics the children live in. More specifically we recommend the dissemination of educational messages on appropriate complementary feeding through popular communication channels like radio, TV or mobile phones interpersonal communications. At community level we recommend the implementation of practical trainings on the preparation of diverse complementary foods, as well as the improvement of the services provided by the nutritional centres in charge of community child growth monitoring.

## Supporting information

S1 Table Variables used for the construction of indices through categorical principal component analysis. (PDF)

S2 Table Factors associated with MDD-C in rural Burundi and rural Rwanda DHS2010. Bivariable models for individual and household variables.
(PDF)

S3 Table Factors associated with MDD-C in rural Burundi and rural Rwanda DHS2010. Bivariable analyses for community variables.
(PDF)
