# Supplementary material for: Children’s dietary diversity and related factors in Rwanda and Burundi: A multilevel analysis using 2010 Demographic and Health Surveys
Source: PLoS One. 2019 Oct 9;14(10):e0223237. doi: 10.1371/journal.pone.0223237 (PMC6785172; doi:10.1371/journal.pone.0223237)
Supplement: S1 Table — (PDF) [file pone.0223237.s001.pdf]

S1 Table: Variables used for the construction of indices through categorical principal component analysis

**Index**	**Variables**
**Educational index**	Mother's educational attainment<br>Literacy level of mother<br>Husband/partner's educational attainment
**Occupation index**	Mother having a job in the last 12 months<br>Mother's occupation<br>Mother employed all year/seasonal<br>Husband/partner's occupation (ocu_partner)
**Living conditions index***	(1) Access to utilities (e.g., water, toilet, fuel, …)<br>(2) Housing conditions (e.g., overcrawding, floor, wall)<br>(3) Possession of durables (e.g., radio, tv and refrigerator)
**Agricultural index**	Livestock owned<br>Agricultural land owned by household (has)

* The living conditions index was constructed with variables from three different dimenssions (1) Access to utilities, (2) Housing conditions and (3) Possession of durables
