# Supplementary material for: Children’s dietary diversity and related factors in Rwanda and Burundi: A multilevel analysis using 2010 Demographic and Health Surveys
Source: PLoS One. 2019 Oct 9;14(10):e0223237. doi: 10.1371/journal.pone.0223237 (PMC6785172; doi:10.1371/journal.pone.0223237)
Supplement: S2 Table — Bivariable models for individual and household variables. (PDF) [file pone.0223237.s002.pdf]

S2 Table: Factors associated with MDD-C in rural Burundi and rural Rwanda DHS2010.Bivariable models for individual and household variables.

COVARIATES		BURUNDI (N=957)			RWANDA (N=1049)		
**Variables**	**Categories**	n	MDDw %	p	**n**	MDDw %	p
**Sex of the child**	**Female**	486	17.7		525	21.9	
	Male	471	13.8	0.098	524	24.1	0.410
**Age of the child**	**6-11 months**	316	11.1		353	15.3	
	12-17 months	324	18.2		342	26.6	
	18-23 months	317	18	0.020	354	27.1	<0.0001
**Mother's age**	**15- 24 years**	263	17.1		276	22.1	
	25-34 years	469	15.1		534	23.8	
	>34 years	225	15.6	0.55	239	22.2	0.82
**Age of respondent at 1st birth**	**12-18 years**	241	18.3		165	13.3	
	19-24 years	629	14.8		702	23.8	
	25-37 years	87	16.1	0.46	182	28.6	0.002
**Mother's education**	**No education**	538	13.2		189	18.5	
	primary	380	18.2		794	22.5	
	secondary and higher	39	28.2	0.01	66	43.9	0.047
**Husband/partner's education**	**No education**	386	11.4		188	19.2	
	Primary	487	17.3		688	21.1	
	Secondary or higher	84	27.4	0.001	173	34.7	<0.0001
**Husband/partner's ocupation**	**Agriculture related**	685	14.1		738	21	
	Other	272	20.2	0.02	311	27.7	0.019
**Age of head of the household**	**Below 31**	427	14.8		429	18.2	
	Between 30 and 40	271	16.6		315	27.6	
	between 40 and 50	169	16.6		186	24.2	
	More than 50	90	16.7	0.894	119	26.1	0.017
**Frequency of listening to radio**	**at least once a week**	644	25.3		519	19.3	
	less than once a week	403	19.4	0.026	437	11.7	0.001
**Number of children under 5 in hh**	**One**	245	14.7		390	26.4	
	Two	542	16.2		531	21.7	
	More than 2	167	15.6	0.858	124	17.7	0.08
**Occupation index**	**Low**	320	13.5		362	27.1	
	Middle	408	14.5		687	20.8	
	High	229	21.4	0.026	374	17.1	<0.0001
**Education index**	**Low**	327	9.5		358	24.3	
	Middle	331	16.6		317	28.4	
	High	299	21.7	<0.0001	350	16.9	0.002
**Durable good index**	**Low**	319	10.3		350	19.4	
	Middle	319	14.1		349	32.7	
	High	319	22.9	<0.0001	371	20	<0.0001
**Agricultural index**	Low	356	13.5		331	19.6	
	Middle	283	16.3		347	29.4	
	High	318	17.9	0.278	350	16.3	0.002
**Wealth index (combined)**	Low	319	9.7		350	19.4	
	Middle	319	14.4		349	33.2	
	High	319	23.2	<0.0001			<0.0001
