# Supplementary material for: Children’s dietary diversity and related factors in Rwanda and Burundi: A multilevel analysis using 2010 Demographic and Health Surveys
Source: PLoS One. 2019 Oct 9;14(10):e0223237. doi: 10.1371/journal.pone.0223237 (PMC6785172; doi:10.1371/journal.pone.0223237)
Supplement: S3 Table — Bivariable analyses for community variables. (PDF) [file pone.0223237.s003.pdf]

S3 Table: Factors associated with MDD-C in rural Burundi and rural Rwanda DHS2010. Bivariable analyses for community variables.

COVARIATES		BURUNDI (N=957)			RWANDA (N=1049)		
Variables	Categories	n	MDDw %	p	n	MDDw %	p
**Altitud (continua)**		957	15.6	0.1	1049	23.0	0.1
**Normlized Difference Vegetation Index**	**0-0.6**	577	14.9		561	24.8	
	0.6-1.00	380	17.1	0.4	488	20.9	0.1
**Population**	**low tertile**	319	16.6		352	21.9	
	med tertile	320	12.5		348	21.8	
	high tertile	318	18.2	0.1	349	25.4	0.1
**Community Endowment Index rescalated**	**low**	319	16.3		352	21.9	
	medium	322	16.2		354	21.8	
	high	316	14.9	0.863	343	25.4	0.863
**Soil Constraints Final**	**No constraints**	0	0.0		83	18.1	
	Partly with constraints	119	13.5		455	25.3	
	Frequent Severe constraints	317	18.0		283	20.9	
	Very frequent severe constraints	521	15.0	0.496	228	22.8	0.36
